# Predictors for diabetic retinopathy in normoalbuminuric people with type 2 diabetes mellitus

**DOI:** 10.1186/1758-5996-4-29

**Published:** 2012-07-02

**Authors:** Ho Ra, Ji Han Yoo, Woo Ho Ban, Ho Cheol Song, Seong Su Lee, Sung Rae Kim, Soon Jib Yoo, Yong-Soo Kim, Euy Jin Choi, Yong Kyun Kim

**Affiliations:** 1 Department of Ophthalmology and Visual Science, College of Medicine, The Catholic University of Korea, Seoul, South Korea; 2 Department of Internal Medicine, College of Medicine, The Catholic University of Korea, Seoul, South Korea; 3 Department of Internal Medicine, Bucheon Saint Mary’s Hospital, Sosa-dong, Wonmi-gu, Bucheon-si, Geoynggi-do, 420-717, South Korea

**Keywords:** Albuminuria, Risk factor, Diabetic retinopathy, Type 2 diabetes mellitus, Hemoglobin

## Abstract

****Background**:**

Previous studies have reported that microalbuminuria is an independent risk factor for the prevalence of diabetic retinopathy (DR) in patients with type 2 diabetes mellitus (DM). For this reason, the clinical significance of DR in normoalbuminuric type 2 DM patients may be overlooked. The aim of this study was to investigate the prevalence of DR and predictors for DR in normoalbuminuric patients with type 2 DM.

****Methods**:**

A total 310 patients with type 2 DM and normoalbuminuria, who were referred to the Department of Ophthalmology for screening of DR were included in this study. DR was clinically graded according to the International Clinical Diabetic Retinopathy guidelines. The urinary albumin excretion rate (UAER) was assessed via 24-hour urine collection and measured by immunoturbidimetric assay. Normoalbuminuria was defined as a UAER < 20 μg/min in 2 out of 3 consecutive tests taken within 2–3 months.

****Results**:**

DR of any grade was present in 64/310 (20.7 %) patients. Mild non-proliferative diabetic retinopathy (NPDR) was most prevalent in patients with DR of any grade (36/64, 56 %). The duration of diabetes (OR 1.01, 95 % CI, 1.01 – 1.02, p < 0.001), hemoglobin levels (OR 0.73, 95 % CI, 0.59 – 0.91, p = 0.004) and a higher tertile of UAER (OR 4.04, 95 % CI, 1.71 – 9.57, p = 0.001) had independently significant association with DR. NPDR as well as PDR was more prevalent in patients with higher tertile of UAER compared with those with lower tertile of UAER (NPDR, p = 0.002 and PDR, p = 0.027, respectively).

****Conclusions**:**

Our findings suggest that patients with normoalbuminuric type 2 DM also require close monitoring for the early detection of DR, especially if they have a higher UAER, longer duration of diabetes, or lower hemoglobin levels.

## **Background**

Diabetic retinopathy (DR) is one of the leading causes of visual impairment and blindness in people with diabetes mellitus (DM) [1-3]. As the prevalence of DM increases, the development of DR as a microvascular complication of DM also rises. Furthermore, previous studies demonstrated that DR has also been associated with cardiovascular and all cause mortality as well as visual morbidity in patients with type 2 DM [4,5], which adds value to investigating the prevalence and risk factors for DR.

Microalbuminuria is a marker of endothelial dysfunction and may influence on alterations in the microvasculature of retina and kidneys. Previous studies have demonstrated that microalbuminuria is predictive for certain diabetic complications such as diabetic nephropathy, and is a useful marker for early detection and treatment [6]. Regarding the association between microalbuminuria and DR, microalbuminuria has been demonstrated to be an independent risk factor for the incidence of DR in patients with type 1 DM [6-9]. However, in patients with type 2 DM, it is controversial whether microalbuminuria is independently associated with the incidence of DR [6,10,11]. These findings suggest that DR might develop in patients with type 2 DM without microalbuminuria. Furthermore, previous studies reported that DR is prevalent in normoalbuminuric patients with type 2 DM and its prevalence is ~10-30 % [11-13]. However, there are few data concerning the prevalence of DR according to the severity and predictors for DR in normoalbuminuric patients with type 2 DM. Establishing the prevalence of DR according to the severity and predictors for DR may be helpful for early detection and treatment of DR in normoalbuminuric patients with type 2 DM. Therefore, the purpose of this study was to investigate the prevalence of DR according to the severity and predictors for DR in normoalbuminuric patients with type 2 DM.

## **Materials and methods**

### **Study population**

This was a retrospective cross-sectional study. The present study included patients with type 2 DM and normoalbuminuria, who were referred to the Department of Ophthalmology for screening of DR and underwent a dilated fundus examination and fundus photographic evaluation at the Bucheon Saint Mary’s Hospital between July 2005 and December 2007. Type 2 DM was diagnosed if the patients with a fasting plasma glucose level ≥126 mg/dl or a 2-h post-glucose level after a 75-g oral glucose tolerance test ≥ 200 mg/dl [14]. Patients who were treated by diet alone or in combination with oral hypoglycemic agents or they had fasting serum C-peptide values greater than 1.0 ng/mL when administered insulin were also categorized as type 2 DM. Normoalbuminuria was defined as a urinary albumin excretion rate (UAER) < 20 μg/min in 2 out of 3 consecutive tests taken within 2–3 months [15]. The exclusion criteria were 1) an age < 18 years or > 80 years, 2) accelerated hypertension 3) pregnancy, 4) malignancies, 5) severely disabled patients such as hepatic failure or heart failure, 6) schizophrenia, 7) goiter, and 8) acute systemic infection. The study protocol was approved by the local ethical committee, and this study was conducted according to the principles of the Declaration of Helsinki.

### **Ophthalmic examination and definition of diabetic retinopathy**

Each participant underwent a comprehensive ophthalmic examination that included automated refraction (RK-F1, Canon), non-contact tonometry (TX-F, Canon), lens grading at the slit lamp, and stereoscopic fundus examination using an indirect ophthalmoscope, and a slit lamp biomicroscope with a “superfield lens” (Volk, Mentor, OH, USA) after pupil dilatation with 1.0 % tropicamide and 10 % phenylephrine. Fundus photographs (45 °) were taken from both eyes of each participant using a digital fundus camera (VX-10i, Kowa, Japan). The photographs were taken in 1-field per eye, centered on the macula. Diagnosis of DR was made by a trained ophthalmologist by an indirect ophthalmoscopic examination based on the presence of clinical features in the fundus of both eyes following the International Clinical Diabetic Retinopathy guidelines, and classified as (i) no apparent retinopathy, (ii) mild nonproliferative diabetic retinopathy (NPDR), (iii) moderate NPDR, (iv) severe NPDR, or (iv) proliferative diabetic retinopathy (PDR) [16].

### **Clinical information and laboratory analysis**

Clinical information was assessed from the written and electronic medical records, and this information included the medical history, current medications and laboratory data. The collected data included age, gender, body mass index (BMI), systolic and diastolic blood pressures, the duration of diabetes, serum creatinine, hemoglobin and albumin levels, total cholesterol and high-density lipoprotein (HDL) cholesterol levels, triglyceride and serum uric acid levels, the UAER, the homeostasis model for insulin resistance (HOMA-IR) score, lipoprotein (a) and high-sensitivity C-reactive protein (hs-CRP) levels. Drug histories in relation to anti-hypertensive agents including renin-angiotensin system (RAS) inhibitors were also evaluated. Fasting venous blood samples were taken for the determination of the serum level of total cholesterol, HDL cholesterol and triglyceride. The UAER was assessed via 24 hour urine collection and measured by immunoturbidimetric assay using Roche/Hitachi MODULAR automated clinical chemistry analyzers (Roche Diagnostic, GmbH, Mannheim, Germany). The BMI was calculated as weight (kg)/height (m^2^). The blood pressure was measured twice, 5 minutes apart, using a random zero sphygmomanometer with the patient seated after 10 minutes of rest. The estimated GFR (eGFR) was calculated using the Modification of Diet in Renal Disease four-variable equation at the time of CT scanning: eGFR = 186xserum creatinine^-1.154^xage^-0.203^x1.212 (if black)x0.742 (if female) [17]. The HOMA-IR was calculated with the following formula: [Fasting insulin (μIU/mL) × fasting plasma glucose (mmol/L)]/22.5 [18].

### **Statistics**

The data for continuous variables with a normal distribution is expressed as means ± SDs and the data for the continuous variables without a normal distribution is expressed as medians with interquartile ranges. The Student’s *t*-test or Mann–Whitney test were used, as appropriate, to determine differences in continuous variables. Categorical variables are presented as percentage. The Pearson’s chi-square test or Fisher’s exact test, as appropriate, was used to determine the differences in categorical variables. Both univariate and multivariate logistic regression analyses were performed to determine the various risk factors for the presence of DR. From the univariate analysis, variables with p < 0.05 and those which were already established as risk factors were included in the multivariate logistic regression analysis. These variables included age, gender, systolic blood pressure, duration of DM, eGFR, hemoglobin, use of RAS inhibitors and urinary albumin excretion rate. *P values* < 0.05 were considered statistically significant. The statistical analyses were performed using SPSS 15.0 software (Chicago, IL, USA).

## **Results**

A total 310 patients were enrolled in this study. The mean age of enrolled patients was 54 ± 14 years (median, 54 years; interquartile range, 46 – 65 years). This study included 153 males (49.4 %) and 157 females (50.6 %). Median duration of diabetes was 29 months (interquartile range, 2 – 110 months) and the mean eGFR was 97 ± 25 ml/min/1.73 m^2^.

Table 1 shows the prevalence of DR according to the diabetic retinopathy staging system presented by the International Clinical Diabetic Retinopathy guidelines. DR of any grade was present in 64/310 (20.7 %) patients. Of the patients with DR of any grade, 54/64 (84.4 %) had NPDR and 10/64 (15.6 %) had PDR. Of the patients with NPDR, 36/54 (66.7 %) had mild NPDR, 5/54 (9.3 %) had moderate NPDR and 13/54 (24.1 %) had severe NPDR, respectively.

**Table 1:** Prevalence of diabetic retinopathy according to the diabetic retinopathy staging system presented by the International Clinical Diabetic Retinopathy guidelines in normoalbuminuric people with type 2 DM

	**Number of patients (n = 310)**
No diabetic retinopathy	246 (79.4)
NPDR, mild	36 (11.6)
NPDR, moderate	5 (1.6)
NPDR, severe	13 (4.2)
PDR	10 (3.2)

Data are expressed as n (%).

Abbreviations: *NPDR* non-proliferative diabetic retinopathy, *PDR* proliferative diabetic retinopathy.

Comparisons of clinical characteristics between the patients with DR and without DR are shown in Table 2. The patients with DR were older than those without DR and DR was more prevalent in females than in males. The patients with DR had lower BMI, higher systolic blood pressure, longer duration of diabetes, lower eGFR, lower hemoglobin levels, lower serum total cholesterol levels and higher UAER compared with those without DR. The number of patients using RAS inhibitors was 101 (32.6 %) and the patients with DR had higher prevalence of using RAS inhibitors compared with those without DR. In univariate logistic regression analysis with the presence of DR as dependent variable, age, gender, BMI, systolic blood pressure, duration of diabetes, eGFR, hemoglobin levels, use of RAS inhibitors and higher tertile of UAER were significantly associated with the presence of DR (Table 3). Multivariate logistic regression analysis to determine the predictor of DR in normolabuminuric people with type 2 DM are shown in Table 4. Data revealed that the duration of diabetes (OR 1.01, 95 % CI, 1.01 – 1.02, p < 0.001), hemoglobin levels (OR 0.73, 95 % CI, 0.59 – 0.91, p = 0.004), and a higher tertile of UAER (OR 4.04, 95 % CI, 1.71 – 9.57, p = 0.001) had independently significant association with DR. As the UAER was a strong predictor of the presence for DR, we analyzed the prevalence of diabetic retinopathy according to the tertiles of urinary albumin excretion rate (Table 5). NPDR as well as PDR was more prevalent in patients with a higher tertile of UAER compared with those with a lower tertile of UAER (NPDR, p = 0.002 and PDR, p = 0.027, respectively).

**Table 2:** Comparisons of clinical characteristics between the patients with DR and without DR

**Clinical characteristics**	**No DR (n = 246)**	**DR (n = 64)**	**P value**
Age, years	53 ± 13	61 ± 12	< 0.001
Female, n (%)	114 (46.3)	43 (67.2)	0.003
BMI, kg/m^2^	25.0 ± 3.7	23.9 ± 3.0	0.022
Systolic blood pressure, mmHg	127 ± 14	132 ± 17	0.018
Diastolic blood pressure, mmHg	77 ± 10	77 ± 9	0.836
Duration of diabetes, months	17 (1–75)	117 (60–191)	< 0.001
Serum creatinine, mg/dL	0.8 ± 0.2	0.8 ± 0.2	0.797
Estimated GFR, ml/min/1.73 m^2^	99 ± 25	89 ± 22	0.003
Hemoglobin, g/dL	13.9 ± 1.8	12.6 ± 1.7	< 0.001
Serum albumin, g/dL	4.4 ± 0.4	4.3 ± 0.4	0.111
Serum total cholesterol, mg/dL	187 ± 42	174 ± 44	0.049
Serum triglyceride, mg/dL	167 ± 119	181 ± 132	0.411
Serum HDL, mg/dL	51 ± 14	48 ± 17	0.076
Serum uric acid, mg/dL	4.3 ± 1.4	4.5 ± 1.6	0.550
Urinary albumin excretion, μg/min	5.92 ± 3.50	8.97 ± 5.07	< 0.001
Hemoglobin A1c	9.2 ± 2.5	9.1 ± 1.9	0.741
HOMA-IR	3.4 ± 4.6	4.0 ± 4.3	0.347
Lipoprotein (a), (IU/L)	135 (46–341)	171 (42–387)	0.496
hs-CRP, mg/dL	0.2 (0.1-0.9)	0.2 (0.1-0.7)	0.854
Use of RAS inhibitors, n (%)	68 (27.6)	33 (51.6)	< 0.001

Values are expressed as means ± SDs, medians (interquartile range) or n(%).

Abbreviations: *DR* diabetic retinopathy, *BMI* body mass index, GFR glomerular filtration rate, *HDL* high-density lipoprotein, *HOMA-IR* homeostasis model for insulin resistance, *hs-CRP* high-sensitivity *C*-reactive protein, *RAS* renin-angiotensin system.

**Table 3:** Univariate logistic regression analysis with diabetic retinopathy as a dependent variable in normolabuminuric people with type 2 DM

	**DR**		
	**Odds ratio**	**95 % CI**	**P value**
Age	1.05	1.03 – 1.08	< 0.001
Gender (female)	2.37	1.33 – 4.23	0.003
BMI	0.91	0.83 – 0.99	0.023
Systolic blood pressure	1.02	1.00 – 1.04	0.019
Diastolic blood pressure	1.00	0.98 – 1.03	0.835
Duration of diabetes	1.01	1.01 – 1.02	< 0.001
Serum creatinine	1.16	0.37 – 3.70	0.797
Estimated GFR	0.98	0.97 – 0.99	0.004
Hemoglobin	0.67	0.57 – 0.79	< 0.001
Serum albumin	0.57	0.29 – 1.14	0.573
Serum total cholesterol	0.99	0.99 - 1.00	0.050
Serum triglyceride	1.00	1.00 – 1.00	0.411
Serum HDL	0.98	0.96 – 1.00	0.077
Serum uric acid	1.06	0.88 – 1.28	0.549
Hemoglobin A1c	0.96	0.88 – 1.07	0.418
HOMA-IR	0.98	0.87 – 1.10	0.981
Lipoprotein (a)	1.0	1.0 – 1.0	0.876
hs-CRP	1.01	0.99 – 1.04	0.375
Use of RAS inhibitors	2.79	1.59 – 4.90	< 0.001
Urinary albumin excretion rate tertiles			
I (< 4.26 μg/min)	1	-	-
II (4.26 -7.21 μg/min)	1.38	0.62 – 3.08	0.433
III (> 7.21 μg/min)	4.08	1.97 – 8.42	< 0.001

Abbreviations: *DR* diabetic retinopathy, *BMI* body mass index, *GFR* glomerular filtration rate, *HDL* high-density lipoprotein, *HOMA-IR* homeostasis model for insulin resistance, *hs-CRP* high-sensitivity *C*-reactive protein, *RAS* renin-angiotensin system.

**Table 4:** Multivariate logistic regression analysis to determine the predictor of diabetic retinopathy in normolabuminuric people with type 2 DM

	**DR**		
	**Odds ratio**	**95 % CI**	**P value**
Age	1.00	0.97 – 1.04	0.872
Gender (female)	1.08	0.51 – 2.28	0.837
Systolic blood pressure	1.02	0.99 – 1.04	0.197
Duration of diabetes	1.01	1.01 – 1.02	< 0.001
Estimated GFR	1.00	0.99 – 1.02	0.985
Hemoglobin	0.73	0.59 – 0.91	0.004
Use of RAS inhibitors	1.83	0.92 – 3.62	0.084
Urinary albumin excretion rate tertiles			
I (< 4.26 μg/min)	1	-	-
II (4.26 -7.21 μg/min)	1.34	0.52 – 3.44	0.543
III (> 7.21 μg/min)	4.04	1.71 – 9.57	0.001

Abbreviations: *DR*, diabetic retinopathy; *GFR*, glomerular filtration rate; *RAS*, renin-angiotensin system.

**Table 5:** Prevalence of diabetic retinopathy by the International Clinical Diabetic Retinopathy guidelines according to the tertiles of urinary albumin excretion rate in normoalbuminuric people with type 2 DM

	**Urinary albumin excretion rate**			**P value**
	**Tertile I(< 4.26 μg/min)n = 103**	**Tertile II(4.26 -7.21 μg/min)n = 104**	**Tertile III(> 7.21 μg/min)n = 103**	
No DR	91 (88.3)	88 (84.6)	67 (65.0)	< 0.001
NPDR	11 (10.7)	14 (13.5)	29 (28.2)	0.002
NPDR, mild	8 (7.8)	8 (7.7)	20 (19.4)	
NPDR, moderate	0 (0)	3 (2.9)	2 (1.9)	
NPDR, severe	3 (2.9)	3 (2.9)	7 (6.8)	
PDR	1 (1.0)	2 (1.9)	7 (6.8)	0.027

Data are expressed as n (%).

Abbreviations: *NPDR* non-proliferative diabetic retinopathy, *PDR* proliferative diabetic retinopathy.

## **Discussion**

In this study, we reported the prevalence of diabetic retinopathy according to the severity and predictors for DR in normoalbuminuric patients with type 2 DM. Previous studies reported that microalbuminuria was independently associated with the prevalence of NPDR and PDR in patients with type 2 DM [11,12,19-21]. For this reason, the clinical significance of DR in normoalbuminuric type 2 DM patients may be overlooked. Normoalbuminuria is much more prevalent than microalbuminuria and macroalbuminuria in patients with type 2 DM. The prevalence of normoalbuminuria was reported to be 68 – 81 % in patients with type 2 DM in population-based cross sectional studies [12,22,23]. In spite of the high prevalence of normoalbuminuria in patients with type 2 DM, the prevalence of diabetic retinopathy according to the severity and predictors for DR in normoalbuminuric patients with type 2 DM has not been well studied.

In our study, the prevalence of DR with any grade was 20.7 %, which is similar to the prevalence of DR as 10 – 30 % reported in previous studies on normoalbuminuric patients with type 2 DM [11-13]. Our study also analyzed the prevalence of DR according to its severity. NPDR was more prevalent than PDR, and mild NPDR was most prevalent in patients with NPDR with any grade. Our findings mean that DR is prevalent even in normoalbuminuric patients with type 2 DM, and mild NPDR is most the common form according to the DR staging system presented by the International Clinical Diabetic Retinopathy guidelines.

In our study, we showed that the duration of diabetes, hemoglobin levels, and UAER were independent predictors for the presence of DR in normoalbuminuric patients with type 2 DM. Interestingly, the UAER was the strongest predictor for the presence of DR in normoalbuminuric patients with type 2 DM even after adjustment for age, gender, systolic blood pressure, duration of DM, eGFR and use of RAS inhibitors. Previous studies have reported that microalbuminuria is associated with the prevalence of DR in patients with type 2 DM [19-21]. Most of the previous studies included type 2 DM patients with not only normoalbuminuria, but also microalbuminuria and macroalbuminuria. It has not been well known whether the UAER could predict the presence of DR even in normoalbuminuric patients with type 2 DM. To our knowledge, this study is the first to investigate the association between UAER and the presence of DR in normoalbuminuric patients with type 2 DM. Furthermore, in our study, not only NPDR, but also PDR, was more prevalent in patients with a higher tertile of UAER compared with those with a lower tertile of UAER, which means that UAER was associated with the severity of DR in normoalbuminuric people with type 2 DM. Our findings suggest that even in patients with normoalbuminuric type 2 DM, close monitoring for DR might be needed for the early detection of DR if they have a higher UAER.

In our study, a longer duration of diabetes and lower hemoglobin levels were also independently associated with the prevalence of DR. The duration of diabetes is a well known predictor for the presence of DR in patients with type 2 DM [12,21,24]. The duration of diabetes is regarded as a marker for long-term exposure to hyperglycemia. Therefore, longer duration of diabetes may contribute the prevalence of DR. Lower hemoglobin levels were an independent predictor for the occurrence and severity of DR in the previous studies [21,25]. There is no clear evidence for the mechanism underlying the association of anemia with DR. However, there are some possible mechanisms of how anemia can adversely affect medical outcomes. Hypoxia may alter angiogenesis, capillary permeability, vasomotor response and cell survival [26], which may contribute the adverse medical outcomes including occurrence and progression of DR. Our findings add value by supporting the use of the duration of diabetes and hemoglobin levels as additional predictors for the presence of DR in patients with normoalbuminuric type 2 DM.

Our study has some limitations. First, this study was a cross-sectional study and the sample size was relatively small. A larger prospective study may further elucidate the occurrence and predictors of DR in patients with normoalbuminuric type 2 DM. Second, this study is single center study; therefore, the generalizability of our results to other ethnic groups with type 2 DM is uncertain.

## **Conclusions**

DR is prevalent even in normoalbuminuric patients with type 2 DM, and mild NPDR is the most common form. The duration of diabetes, hemoglobin levels, and UAER were independent predictors for the presence of DR, and the UAER was the strongest predictor for DR. Our findings suggest that even in patients with normoalbuminuric type 2 DM, close monitoring for DR might be needed for early detection of DR, especially if they have a higher UAER, longer duration of diabetes, or lower hemoglobin levels.

## **Competing interests**

The authors declare that they have no competing interests.

## **Author’s contributions**

HR, JHY, WHB, HCS, SSL, SRK, SJY, YSK, and EJC contributed in execution, analysis and manuscript drafting. YKK contributed in study design, execution, analysis, manuscript drafting and critical discussion. All authors read and approved the final manuscript.
